# Invasive Coronary Assessment in Myocardial Ischemia with No Obstructive Coronary Arteries

**DOI:** 10.1007/s11883-023-01144-9

**Published:** 2023-09-08

**Authors:** Tatsunori Takahashi, Aakriti Gupta, Bruce A. Samuels, Janet Wei

**Affiliations:** 1 Smidt Heart Institute, Cedars-Sinai Medical Center, Los Angeles, CA USA; 2 Barbra Streisand Women’s Heart Center, Smidt Heart Institute, Cedars-Sinai Medical Center, 127 S San Vicente Blvd A3212, Los Angeles, CA 90048 USA

**Keywords:** INOCA, Coronary microvascular dysfunction, Vasospastic angina, Invasive coronary function testing

## Abstract

**Purpose of Review:**

The purpose of this review is threefold: (i) to give an overview of well-established invasive methods for assessing patients with ischemia with no obstructive coronary arteries (INOCA) in the cardiac catheterization laboratory; (ii) to describe the prognostic and treatment implications based on these findings, and (iii) to discuss current knowledge gaps and future perspectives.

**Recent Findings:**

Recent studies have demonstrated that invasive coronary function testing not only allows for risk stratification of patients with INOCA but also guides medical therapy with improvement in symptoms and quality of life. Based on these findings, invasive coronary function assessment is now a class 2a recommendation in the 2021 ACC/AHA chest pain guideline to improve the diagnosis of coronary microvascular dysfunction and to enhance risk stratification.

**Summary:**

Invasive functional testing for patients with INOCA is well established and easily performed in the catheterization laboratory. Comprehensive invasive assessment is a key to differentiating INOCA endotypes and optimizing both medical therapy and preventive strategies including lifestyle modification.

## Introduction

Patients with angina and/or signs of ischemic heart disease are often found to have no obstructive coronary artery disease (CAD) [1]. This condition is referred to as ischemia with no obstructive coronary arteries (INOCA) and affects up to 70% of women [2] and up to 50% of men [3]. INOCA is a heterogeneous and non-benign condition associated with poor cardiovascular outcomes and economic burden [1, 4, 5]. Thus, it is imperative to identify those at risk and optimize therapy [6]. Invasive coronary function testing offers a distinctive advantage over non-invasive assessments due to a comprehensive assessment of microvascular and vasospastic angina (Table 1) [7, 8], and is currently a class 2a recommendation for patients with suspected INOCA in the latest American College of Cardiology (ACC) and American Heart Association (AHA) chest pain guideline [9••].

**Table 1:** Diagnostic criteria for microvascular angina and vasospastic angina by the Coronary Vasomotion Disorders International Study Group

Criteria	Microvascular angina	Vasospastic angina
1. Symptoms of myocardial ischemia	(a) Effort and/or rest angina (b) Angina equivalents (i.e., shortness of breath)	Nitrate-responsive angina during spontaneous episode, with at least one of the following: (a) Rest angina - especially between night and early morning (b) Marked diurnal variation in exercise tolerance - reduced in morning (c) Hyperventilation can precipitate an episode (d) Calcium channel blockers (but not beta-blockers) suppress episodes
2. Absence of obstructive CAD (<50% diameter reduction or FFR<0.80)	(a) Coronary CTA (b) Invasive coronary angiography	(a) Coronary CTA (b) Invasive coronary angiography
3. Objective evidence of myocardial ischemia	(a) Ischemic ECG changes during an episode of chest pain (b) Stress-induced chest pain and/or ischemic ECG changes in the presence or absence of transient/reversible abnormal myocardial perfusion and/or wall motion abnormality	Transient ischemic ECG changes during spontaneous episode, including any of the following in at least two contiguous leads: (a) ST segment elevation ≥0.1 mV (b) ST segment depression ≥0.1 mV (c) New negative U waves
4. Evidence of coronary dysfunction	(a) Impaired coronary flow reserve (cut-off values depending on methodology use between ≤2.0 and ≤2.5) (b) Coronary microvascular spasm, defined as reproduction of symptoms, ischemic ECG shifts but no epicardial spasm during acetylcholine testing. (c) Abnormal coronary microvascular resistance indices (e.g., IMR >25) (d) Coronary slow flow phenomenon, defined as TIMI frame count >25.	Coronary artery spasm defined as transient total or subtotal coronary artery occlusion (>90% constriction) with angina and ischemic ECG changes either spontaneously or in response to a provocative stimulus (typically acetylcholine, ergot, or hyperventilation)

“Definitive” if all four criteria are present while “suspected” if criteria 1 and 2 are met but only criteria 3 or 4 is present or equivocal. *ECG* Electrocardiogram, *CAD* coronary artery disease, *CTA* computed tomographic angiography, *FFR* fractional flow reserve, *IMR* index of microcirculatory resistance, *TIMI* thrombolysis in myocardial infarction

Due to a growing recognition of INOCA and technical advances in diagnostic testing, multiple studies have proposed and validated various invasive approaches for the differentiation of INOCA endotypes, prognostication, and individualization of therapy in recent years. The current review gives an overview of each well-established invasive assessment and discusses prognostic value and treatment implications in INOCA.

## Structure and Function of the Coronary Circulation

**Fig. 1:**
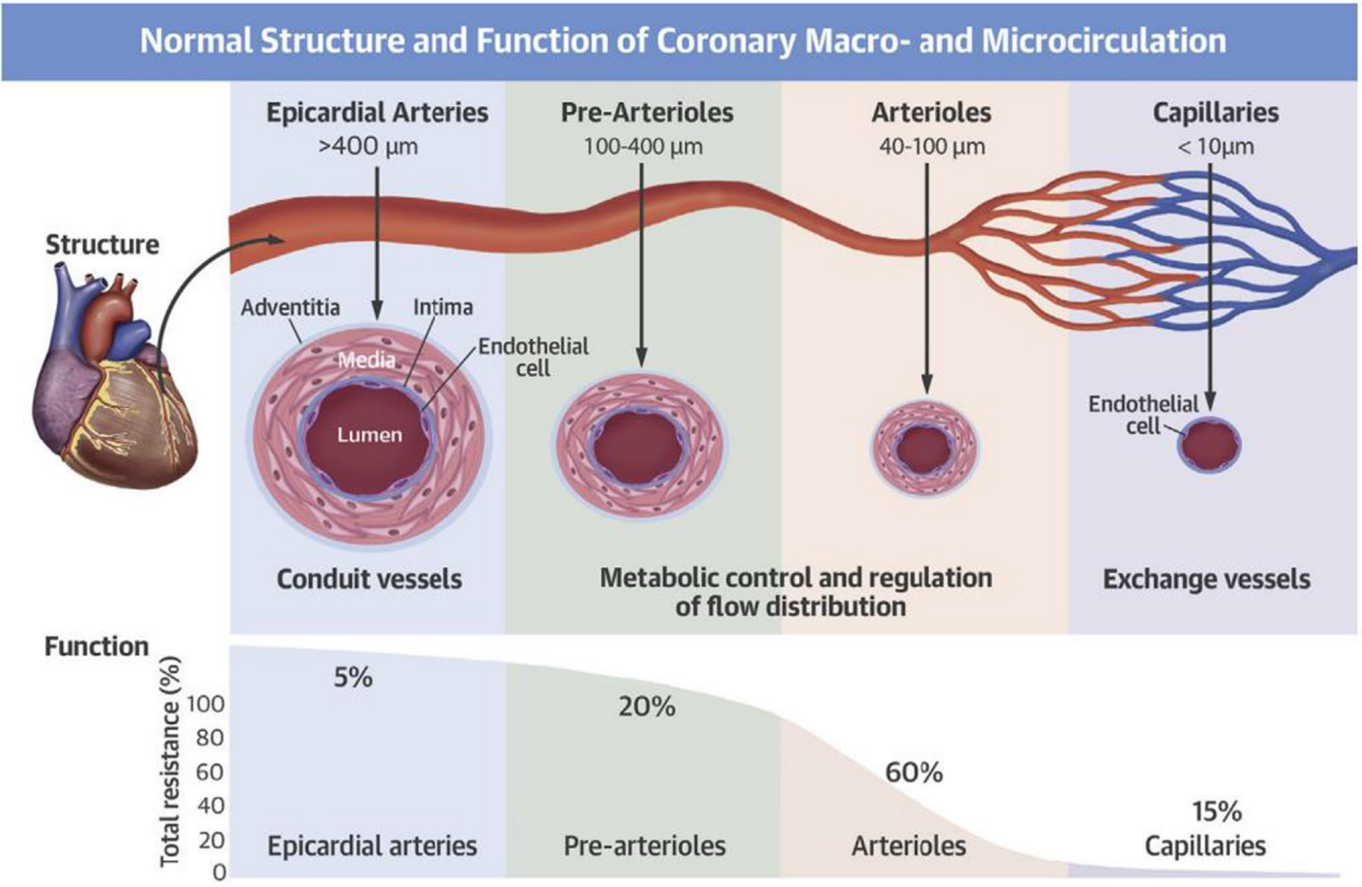
Normal structure and function of coronary macrocirculation and microcirculation. (Reprinted from Taqueti and Di Carli^10^ with permission. Copyright ©2018, Elsevier)

The coronary arterial system consists of the epicardial coronary artery (>400 μm), pre-arterioles (100–400 μm), and arterioles (40–100 μm) each of which is regulated by different mechanisms (Fig. 1) [10]. The epicardial vessels represent 5–10% of the total coronary vascular resistance and are responsive to flow-dependent dilatation [11]. Pre-arterioles, especially distal pre-arterioles, are more sensitive to intravascular pressure variations whereas arterioles are responsive to changes in the intramyocardial concentration of metabolites [11]. The pre-arterioles and arterioles account for most of the total vascular resistance (80%) and control coronary artery blood flow by endothelium-dependent and -independent mechanisms. The normal coronary endothelium releases various vasodilatory or vasoconstrictive substances, which, in turn, affect the tone of vascular smooth muscle cells [12]. In contrast, the myogenic response of arterioles is independent of the coronary endothelium and plays a key role in maintaining coronary microvascular tone in response to pressure changes [13].

Adenosine and acetylcholine (ACh) are the two vasoactive agents most used in the cardiac catheterization laboratory and act on the coronary arterial system differently. Adenosine primarily induces endothelium-independent relaxation of vascular smooth muscle cells [14, 15], whereas the vasodilatory effect of ACh results from the endothelium-mediated release of vasodilatory substances such as nitric oxide [16, 17]. ACh also has a vasoconstrictive effect via direct vasoconstriction of vascular smooth muscle cells especially at a higher ACh dose [16].

## Limitations of Traditional Cardiovascular Testing and Angiography

Traditional cardiovascular testing, such as exercise treadmill testing, stress echocardiography, and SPECT, have limited diagnostic accuracy for detecting coronary vasomotor dysfunction (a reported sensitivity and specificity of non-invasive testing are 41% and 57%, respectively) [18, 19]. Non-invasive assessments that include myocardial blood flow reserve (MBFR) measurements using stress positron emission tomography (PET) or stress cardiac magnetic resonance (CMR) imaging can detect coronary microvascular dysfunction (CMD) and enhance risk stratification in patients with suspected INOCA [20–25]. As a result, these noninvasive measures of MBFR are also class 2a recommendations for the evaluation of INOCA in the latest chest pain guideline [9••]. However, coronary artery spasm is highly prevalent in INOCA populations [26•], and there is currently no established non-invasive method to diagnose coronary artery spasm accurately and safely.

**Fig. 2:**
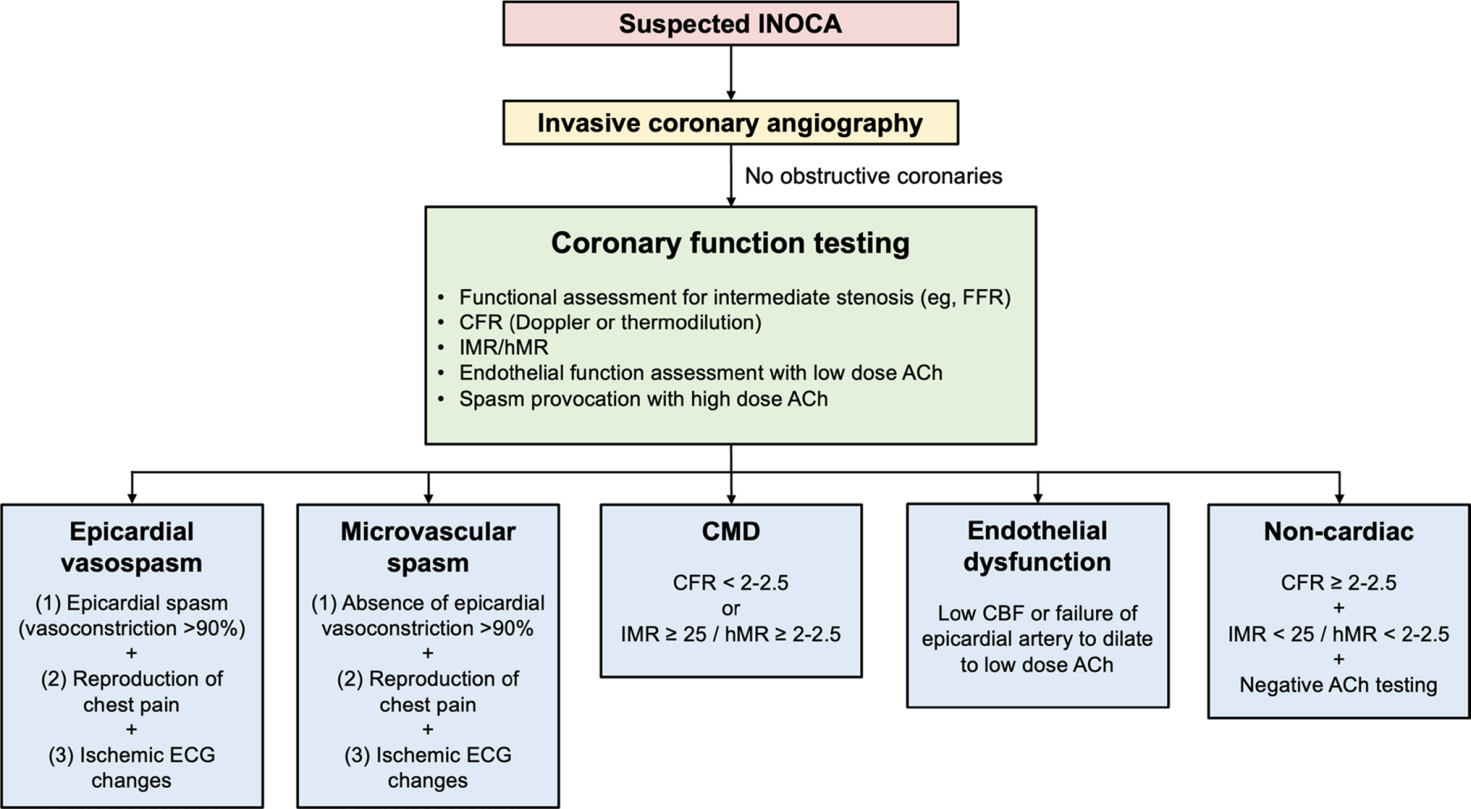
Invasive coronary assessment in INOCA. ACh, acetylcholine; CBF, coronary blood flow; CFR, coronary flow reserve; CMD, coronary microvascular dysfunction; FFR, fractional flow reserve; hMR, hyperemic microvascular resistance; IMR, index of microcirculatory restriction; INOCA, ischemia and no obstructive coronary artery disease

Invasive coronary angiography and coronary computed tomography angiography are the mainstay for the anatomic assessment of CAD and are required to confirm the absence of no hemodynamically significant obstructive stenosis. Moreover, coronary computed tomography angiography is the gold standard for the identification of myocardial bridging [27]. However, the coronary microvasculature is beyond their resolution, limiting structural, and functional assessments of the whole coronary arterial system. Invasive coronary function testing addresses these limitations and allows a more comprehensive assessment of INOCA (Fig. 2).

## Invasive Testing of Coronary Flow Reserve

### Doppler Method

**Fig. 3:**
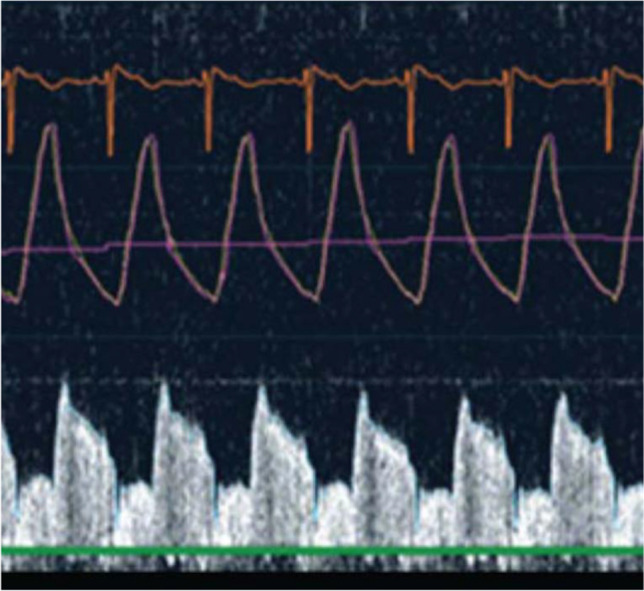
Doppler wire tracing. A Doppler-tipped guidewire allows direct measurement of coronary flow velocity. Doppler peak flow velocities are averaged over three consecutive heartbeats at rest and during hyperemia to calculate resting and hyperemic average peak velocities, respectively

Direct measurement of coronary flow velocity using a Doppler-tipped guidewire is one of the established techniques for the invasive assessment of coronary flow reserve (CFR) (Fig. 3). Peak flow velocities over three consecutive heartbeats are averaged at rest and during hyperemia induced by intravenous or intracoronary administration of adenosine, and Doppler-derived CFR is calculated as the ratio of hyperemic to resting average peak velocity. This technique has existed since the 1970s and may be limited by the ability to obtain a stable, high-quality Doppler flow signal [28, 29]. However, repositioning the Doppler wire helps enable reproducible measurements [30]. It should also be noted that CFRs in response to intravenous infusion of adenosine may result in lower values than intracoronary adenosine injection and caution is needed to interpret CFR depending on how hyperemia is induced [31].

The use of Doppler-derived CFR in INOCA was first validated in a cohort from the WISE (Women’s Ischemia Syndrome Evaluation) study [32]. Doppler-derived CFR in response to intracoronary adenosine infusion was well correlated with coronary volumetric flow reserve, the traditional standard for CMD diagnosis, and demonstrated a high diagnostic performance to identify women with CMD (sensitivity and specificity were 90% and 89%, respectively) [32]. Doppler-derived CFR also has a strong correlation with PET-derived CFR [33], and a widely used cutoff suggestive of CMD in INOCA is <2–2.5 [34–36].

#### Thermodilution Method

**Fig. 4:**
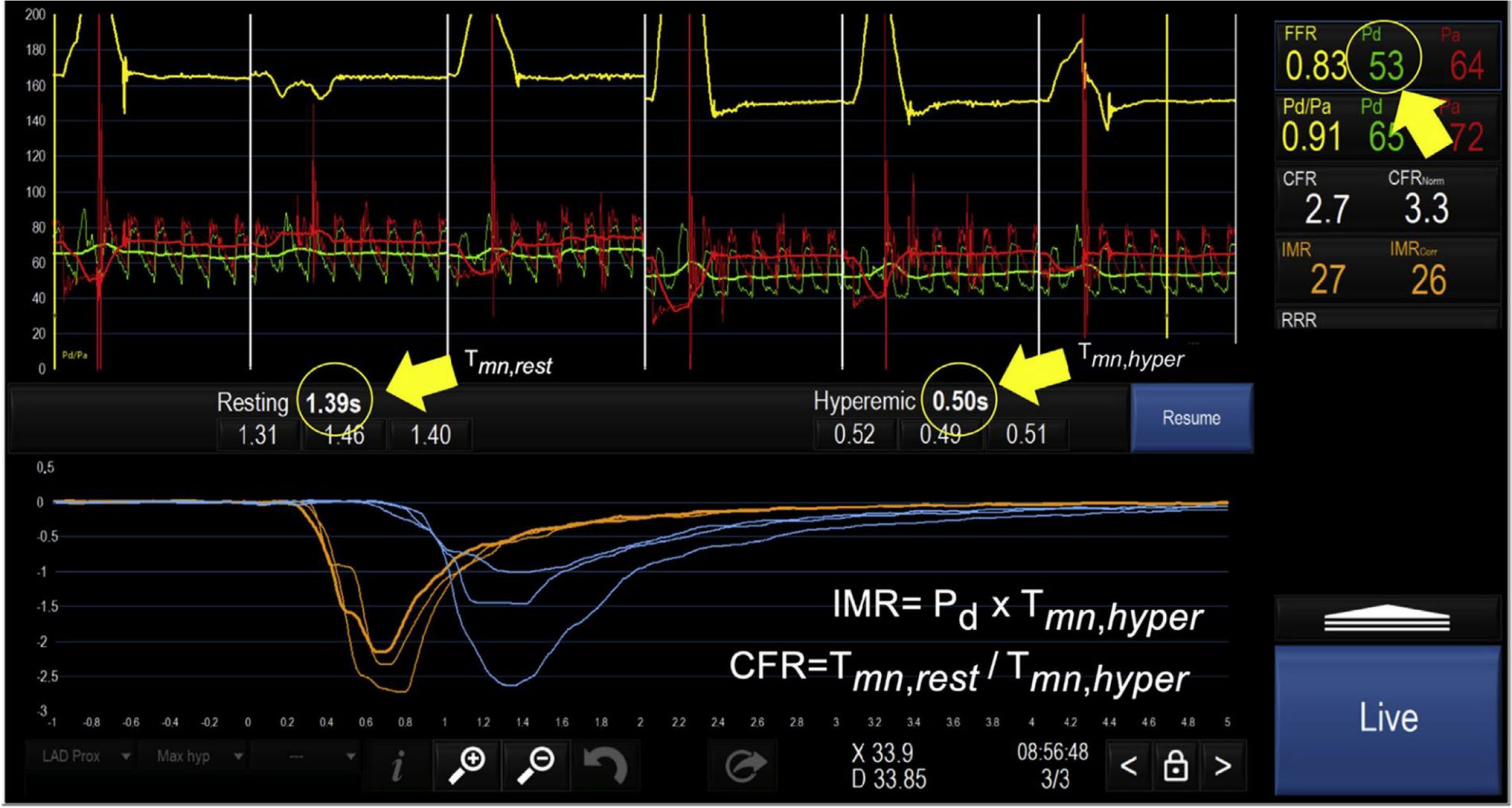
Coronary physiology assessment using the thermodilution technique. Simultaneous recordings of aortic pressure (Pa; red tracing), distal coronary pressure (Pd; green tracing), their ratio (Pd/Pa; yellow tracing), and intracoronary temperature after consecutive injections of 3 boluses at rest (blue tracings) and 3 boluses during steady-state hyperemia (orange tracings). These recordings allow simultaneous measurement of fractional flow reserve (FFR), coronary flow reserve (CFR), and the index of microvascular resistance (IMR). The yellow arrows point to the average values of resting and hyperemic mean transient time (Tmn) as well as to the average distal coronary pressure (Pd) during hyperemia. These values are needed to derive CFR and IMR. (Reprinted from Candreva et al.^37^ with permission. Copyright ©2021, Elsevier)

CFR can also be estimated using the thermodilution technique, which uses a pressure-temperature sensor-tipped guidewire to monitor temperature changes in response to intracoronary saline administration (Fig. 4) [37]. Room-temperature saline is given as a manual bolus to measure the transit time of the injected saline traveling from the proximal to the distal sensor at rest and during maximal hyperemia induced by intravenous adenosine infusion [38, 39]. This bolus thermodilution method allows indirect measurement of coronary flow velocity and is now the most widely used alternative to the Doppler technique. On the other hand, a newer method in which saline is continuously administered using a dedicated monorail catheter connected to an infusion pump has also emerged [40], allowing direct volumetric blood flow measurement. CFR obtained from this continuous thermodilution technique has a higher reproducibility than bolus thermodilution-derived CFR [41]. It should be noted that the catheter used in this method is available only in some countries and is not approved by the US Food and Drug Administration.

To date, a number of studies have assessed the correlation between CFRs derived from bolus thermodilution, Doppler, and non-invasive techniques [29, 33, 39, 42–44]. These investigations report varying degrees of correlation. The same CFR range as used in the Doppler method (i.e., 2–2.5) is widely adopted for the diagnosis of CMD using thermodilution techniques [4, 36].

### Prognosis and Treatment Implications

Aside from the diagnosis of CMD based on CFR, it is important to recognize that CFR is a continuous predictor of adverse outcomes rather than a step-like threshold [45]. In the WISE study, low CFR (<2.32) was associated with an increased risk of combined death, myocardial infarction, stroke, or congestive heart failure, supporting the usefulness of invasive CFR measurement for risk stratification in women with suspected INOCA [34, 46]. The use of beta-blockers is the standard therapy for patients with a low CFR and was one of the treatment pathways studied in a blinded, randomized fashion in the CorMicA (coronary microvascular angina) trial which demonstrated significant improvement in angina and quality of life in INOCA with medical therapy guided by invasive coronary function testing including CFR measurement [6].

## Invasive Testing of Microvascular Resistance

### Index of Microcirculatory Resistance

The index of microcirculatory resistance (IMR) is calculated as hyperemic mean distal intracoronary pressure multiplied by hyperemic mean transit time both of which can be measured using a pressure-temperature sensor-tipped guidewire in a similar manner to bolus thermodilution-derived CFR (Fig. 4) [47]. However, unlike CFR which assesses the flow status of the entire coronary arterial system, IMR specifically interrogates the coronary microcirculation [47]. Moreover, IMR has better reproducibility and less hemodynamic dependence than CFR [48], addressing limitations inherent to invasive CFR measurement. Recently, efforts have been made to estimate IMR without the need for intracoronary wiring or hyperemia by leveraging techniques for computational physiology based on coronary angiography images [49, 50]. This novel technique has the potential to help streamline comprehensive physiological assessment in the cardiac catheterization laboratory once further validated.

Based on prior studies in healthy subjects [51–53], the normal range of IMR is <25. With this cutoff value, the reported prevalence of CMD in INOCA is approximately 20–40% [54–56]. Although the diagnosis of CMD can be made if CFR is <2.0 in the absence of occlusive epicardial coronary arteries regardless of IMR values in the 2021 AHA/ACC guideline [9••], some patients have discordant results between CFR and IMR (i.e., low CFR and low IMR) due to elevated resting coronary flow [57]. Currently, the underlying mechanisms of discordant results are not fully understood, but concurrent measurement of IMR and CFR is useful to differentiate such an endotype from patients with concordant results (low CFR and high IMR) who appear to be pathophysiologically different [57–59].

### Hyperemic Microcirculatory Resistance

Alternatively, microvascular resistance can be assessed with hyperemic microvascular resistance (hMR), defined as the ratio of hyperemic mean distal pressure to hyperemic average peak velocity, using a pressure/Doppler sensor–tipped guidewire [60]. hMR >2.5 mmHg/cm/s is a common cutoff to diagnose CMD [43, 58, 61]. The correlation of hMR with IMR is modest and hMR may have better diagnostic performance to predict invasive CFR <2.0 than IMR [43, 44].

### Prognosis and Treatment Implications

Evidence suggests that high IMR/hMR accompanied by low CFR or vasospastic angina, but not high IMR/hMR alone, is associated with long-term cardiovascular events in INOCA [59, 62]. This highlights the importance of comprehensive physiological assessments for better risk stratification. As described above, IMR/hMR allows us to identify patients with low CFR due to elevated resting coronary blood flow for who enhancing vasodilation is less likely to work, though vasodilators such as calcium channel blockers are often initiated empirically after being found to have no obstructive CAD [63]. IMR was also part of invasive coronary function testing in the CorMicA trial and helped to optimize stratified medical therapy [6].

## Invasive Testing of Coronary Endothelial Dysfunction

### Coronary Endothelial Dysfunction

ACh-mediated increase in coronary blood flow volume measured using a Doppler-tipped guidewire is a surrogate of coronary endothelial microvascular function [64]. Coronary endothelial dysfunction is defined as [1] <50% increase in coronary blood flow in response to ACh compared with baseline or [2] any degree of epicardial vasoconstriction [64]. The dose of ACh used for the assessment of endothelial function varies in the literature from 20 to 40ug and is generally given as a slower infusion (e.g., over 2 min). This is a lower dose than that in spasm provocation testing as discussed below. It should be noted that normal epicardial endothelial function does not necessarily indicate normal microvascular endothelial function and vice versa [35]. A combination of ACh-mediated coronary blood flow increase and epicardial vasoconstriction assessment is needed for a comprehensive evaluation of coronary endothelial function.

Impaired endothelial function in INOCA as assessed with coronary blood flow response was first reported in the early 1990s and is known to be highly prevalent [65, 66•, 67]. In the WISE study, 58% of women with INOCA were found to have epicardial vasoconstriction in response to ACh [68], highlighting a high prevalence of impaired endothelial macrovascular function in INOCA. Another prior comprehensive investigation has also demonstrated that severe epicardial endothelial dysfunction is common and present with other coronary abnormalities such as CMD and myocardial bridging in INOCA [54]. Such an overlap of possible causes of chest pain illustrates the clinical relevance of comprehensive invasive assessment in those with suspected INOCA.

Although the current ACC/AHA guideline does not specifically recommend invasive coronary endothelial function testing [9••], the assessment of coronary blood flow volume in response to ACh increases the diagnostic yield of coronary function testing. For example, endothelial dysfunction was present in 68% of patients with normal CFR and no inducible coronary artery vasospasm, which was similarly high in patients (80%) with positive spasm testing and/or impaired adenosine-mediated vasodilation [18, 66]. These results support its diagnostic role in INOCA.

### Prognosis and Treatment Implications

Impaired coronary vascular response to ACh is independently associated with an increased risk of cardiovascular events as well as decreased time free of adverse events in patients with INOCA [64, 69]. Endothelial dysfunction is believed to precede clinical coronary atherosclerosis, and the initiation of medications targeting atherosclerotic disease such as angiotensin-converting enzyme inhibitors and statins along with lifestyle modification is recommended in patients with endothelial dysfunction [70, 71].

## Invasive Testing of Coronary Artery Spasm

### Epicardial Vasospasm

**Fig. 5:**
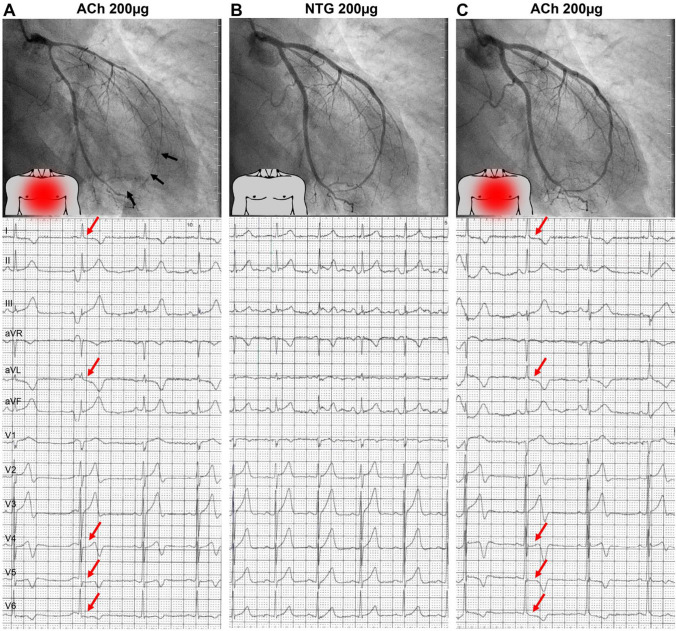
A representative case with positive provocative spasm testing. An example of a patient with **A** diffuse epicardial spasm of the left anterior descending artery (black arrows) provoked by intracoronary injection of 200μg acetylcholine (ACh) accompanied by recognizable chest pain (red dot on torso) and new-onset ischemic ECG changes (red arrows). **B** Coronary spasm, angina symptoms, and ECG changes resolved after intracoronary nitroglycerin (NTG) injection at the end of routine ACh testing. **C** After rechallenge with 200μg ACh 3 minutes later, no epicardial vasoconstriction was observed. However, the patient again reported recognizable chest pain, and ischemic ECG changes (red arrows) reoccurred, suggesting coexisting microvascular spasm refractory to NTG pre-treatment. (Reprinted from Seitz et al.^73^ with permission. Copyright ©2022, Elsevier)

ACh is also used for the assessment of epicardial and microvascular vasospastic angina (VSA) [7]. Invasive protocols to assess coronary vasospasm vary in both dosing and speed of administration [26•]. Dosing is generally incremental, with lower doses used not only to allow for the assessment of endothelial function but also to avoid giving higher doses if epicardial spasm is present at lower doses. The most commonly used dose for assessing epicardial spasm is 100μg, though some groups advocate giving 200μg if spasm is strongly suspected and not present at lower doses. ACh can be manually infused into the coronary artery. Most centers have recommended ACh injection over 30–60 s, but a slower injection of 2 min is adopted in some institutions. Administration should be performed under continuous monitoring of the patient’s symptoms and the 12-lead electrocardiogram [26•]. Intracoronary ACh injection has a high diagnostic performance for the diagnosis of VSA (sensitivity and specificity are 90% and 99%, respectively) [72]. Alternatively, intracoronary ergonovine can be used [7]. Provocative testing is positive if all of the following are induced in response to provocative stimuli; (i) transient total or subtotal coronary artery occlusion (≥ 90% vasoconstriction), (ii) development of chest pain, and (iii) ischemic ECG changes (Fig. 5) [7, 73].

The reported prevalence of VSA is approximately 40% in INOCA [56]. Identifying these patients is crucial because, compared to other endotypes of INOCA, VSA has more established treatment as described below and vasospasm can be prevented in those with an identifiable trigger (e.g., smoking).

### Microvascular Vasospasm

**Fig. 6:**
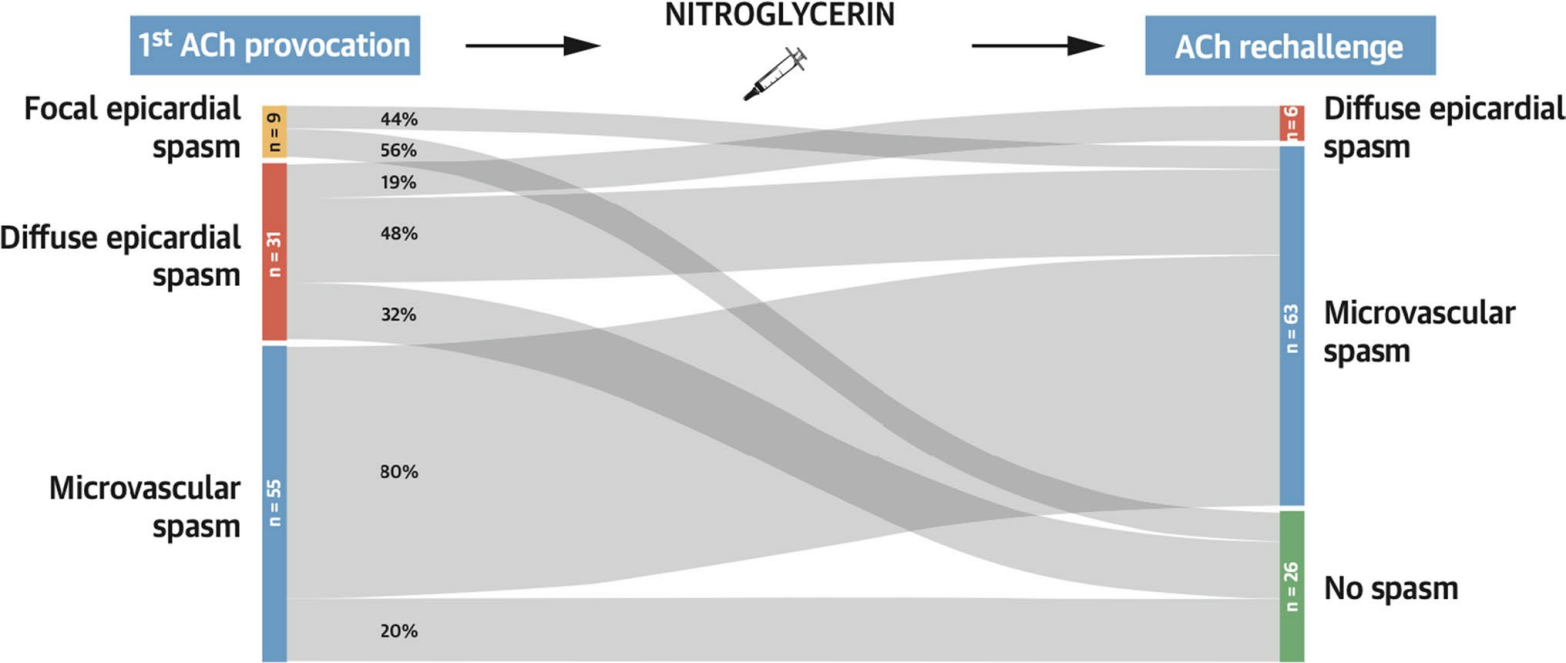
Acetylcholine rechallenge. A Sankey plot illustrating the results of the initial acetylcholine (ACh) spasm provocation test and the ACh rechallenge after nitroglycerin. Nitroglycerin was most effective in preventing focal epicardial spasm and least effective in patients with microvascular spasm. ACh rechallenge revealed coexisting nitroglycerin-refractory microvascular spasm in approximately 50% of patients with focal or diffuse epicardial spasm. (Reprinted from Seitz et al.^73^ with permission. Copyright ©2022, Elsevier)

Microvascular vasospasm (MVS) is defined as symptom reproduction with ischemic ECG changes but no epicardial vasospasm during provocative spasm testing [8]. Although the reported prevalence of MVS ranges from 20 to 40% in INOCA [26•], MVS was traditionally underrecognized because the presence of epicardial spasm during spasm testing masked the diagnosis of MVS. However, a novel method called ACh rechallenge was recently proposed to help detect coexisting MVS with VSA [73]. In this approach, the ACh dose that induced vasospasm is reinjected after intracoronary administration of nitroglycerin. The investigators reported that approximately 50% of patients who were diagnosed with VSA during the initial ACh testing were found to have coexisting MVS after nitroglycerin injection (Figs. 5 and 6) [73]. This result also indicates that nitroglycerin alone may not be adequate to treat such a subset of patients given the remaining microvascular spasm even after nitroglycerin. ACh rechallenge is thus not only diagnostic but also allows us to assess their responsiveness to nitroglycerin.

### Prognosis and Treatment Implications

Recent, long-term follow-up data in a total of 736 INOCA patients who underwent ACh testing demonstrated that the risk of all-cause and cardiac deaths was low irrespective of spasm provocation results [74]. However, VSA was independently associated with myocardial infarction and repeat coronary angiography while patients with MVS were at increased risk of recurrent angina [74]. Although the use of nitrates and calcium channel blockers are the mainstay of pharmacological vasospasm management along with preventive measures (e.g., smoking cessation) [4], nitrates may not be as effective in MVS as in VSA as discussed above [73]. In addition, a recent, randomized, placebo-controlled trial showed no significant improvement in symptoms in patients with MVS treated with diltiazem [75], highlighting challenges in the management of MVS compared to VSA.

## Coronary Slow Flow

### TIMI Frame Count

The coronary slow flow phenomenon is an angiographic finding first reported in 1972 and characterized by the delayed flow of contrast medium in the absence of obstructive CAD [76]. The TIMI (thrombolysis in myocardial infarction) frame count is a semi-quantitative method to assess the degree of slow flow and is determined as the number of cine frames required for contrast to reach the end-point branch [77]. In the international standardization of diagnostic criteria for microvascular angina proposed by the Coronary Vasomotion Disorders International Study Group (COVADIS), a TIMI frame count >25 is listed as evidence of impaired coronary microvascular function along with decreased CFR, high IMR, and MVS [8]. An advantage of the TIMI frame count is no need for wire instrumentation or provocative stimuli. Moreover, previous studies showed that patients with this phenomenon had increased resting microvascular resistance without decreased CFR or elevated hMR [78, 79], which indicates the unique nature of this condition and supports the additive value of the TIMI frame count for identifying patients with microvascular angina who might not be diagnosed by other methods.

### Prognosis and Treatment Implications

The prognostic value of the TIMI frame count was reported in a pilot study from the WISE study [80]. Although the TIMI frame was not predictive of all-cause or cardiovascular mortality, it was an independent predictor of hospitalizations for angina.

A variety of agents such as dipyridamole and simvastatin were studied as a potential pharmacologic intervention for the coronary slow flow phenomenon and were found to improve coronary flow as assessed with the TIMI flow count [81, 82]. However, due to a lack of clinical outcome data, there is currently no established treatment specific to this phenomenon.

## Safety of Coronary Function Testing

The risk of invasive coronary assessment is reported to be low. In the WISE study, serious adverse events related to comprehensive invasive testing occurred in 2 out of 293 women with INOCA (0.7%) and included 1 iatrogenic coronary artery dissection and 1 ST-segment elevation myocardial infarction due to coronary artery spasm [68]. A recent meta-analysis examined the safety of provocative spasm testing with intracoronary ACh and reported excellent safety records in Western populations primarily presenting with INOCA or myocardial infarction with nonobstructive coronary arteries (the rate of procedure-related major complications was 0.0%; 95% confidence interval: 0.0–0.45%) [26•]. The addition of invasive coronary assessment to diagnostic left heart catheterization, which is also a safe procedure in the contemporary era [83], does not appear to significantly increase procedural risk in INOCA.

## Knowledge Gaps and Future Directions

It is currently believed that the additional time and cost related to invasive coronary assessment is offset by preventing unnecessary testing/treatment and future adverse clinical events as a consequence of optimal management guided by invasive testing [84]. However, due to multiple invasive methods available as well as emerging techniques as described above, invasive procedure protocols vary from institution to institution. Thus, it is crucial to develop a standardized, effective protocol from the perspective of time- and cost-efficiency and diagnostic accuracy. Additionally, depending on the endotypes of INOCA, the current management may not be specific but rather be a generalized approach such as risk and lifestyle modification. This highlights the need for therapeutic advances that target underlying pathophysiology and improve long-term clinical outcomes. Rigorous prospective studies are needed to address these knowledge gaps related to invasive diagnosis and subsequent management of INOCA.

## Conclusion

Although obstructive CAD is only one of the many causes of myocardial ischemia, false reassurance after being found no obstructive CAD is often given to patients who might have other structural and/or functional abnormalities in the coronary arterial system. Consequently, these patients continue to have angina with an increased risk of adverse clinical events. INOCA should be recognized as a clinically important diagnosis for which further invasive coronary assessment is indicated to differentiate its endotypes, risk stratify patients, and individualize treatment. This guideline-directed approach needs to be implemented in our clinical practice so that patients with INOCA will receive symptomatic and prognostic benefits.

## Abbreviations

ACh: Acetylcholine
CAD: Coronary artery disease
CFR: Coronary flow reserve
CMD: Coronary microvascular dysfunction
CorMicA: Coronary microvascular angina
COVADIS: Coronary Vasomotion Disorders International Study Group
hMR: Hyperemic microvascular resistance
IMR: The index of microcirculatory resistance
INOCA: Ischemia with non-obstructive coronary arteries
MVS: Microvascular spasm
PET: Positron emission tomography
TIMI: Thrombolysis in myocardial infarction
VSA: Vasospastic angina
WISE: Women’s Ischemia Syndrome Evaluation
